# Dimeric Benzodiazepines as Peptide Mimetics to Overcome p53-Dependent Drug Resistance of Tumors

**DOI:** 10.3390/biom13020291

**Published:** 2023-02-03

**Authors:** Elżbieta Speina, Marcin Wilczek, Adam Mieczkowski

**Affiliations:** 1 Institute of Biochemistry and Biophysics, Polish Academy of Sciences, Pawinskiego 5a, 02-106 Warsaw, Poland; 2 Department of Chemistry, University of Warsaw, Pasteura 1, 02-093 Warsaw, Poland

**Keywords:** p53 protein, drug resistance, cytotoxicity, benzodiazepines, selectivity

## Abstract

Benzodiazepines that consist of one α- and one β-amino acid residues linked together in a seven-membered heterocyclic ring could be treated as small, rigid, cyclic dipeptides capable of exhibiting a wide range of biological activities. During our research on novel analogues of anthramycin, a tricyclic antibiotic benzodiazepine, we developed the synthesis of two benzodiazepine dimers, obtained through the cyclization of appropriate linear tripeptides. The synthesized compounds were tested on a panel of seven cancer and normal cell lines. The developed molecules exhibited promising cytotoxic activity against the lung cancer cell lines A549 and NCI-H1299 and the epidermoid carcinoma cell line A-431. Moreover, they showed significant selectivity compared to the reference cell lines (BJ—human normal skin fibroblasts and MRC-5—human normal lung cell line). When tested on two isogenic cell lines, HCT116 and HCT116p53^−/−^ (colon cancer), contrary to cisplatin being used as a positive control, the obtained compounds showed a cytotoxic effect independent of the p53 protein status. For the above reasons, the obtained compounds can be considered a new group of promising anticancer agents, useful in the fight against p53-dependent drug resistance in cancers. They can also be treated as convenient, leading structures suitable for further optimization and searching for more active and selective molecules.

## 1. Introduction

**Figure 1:**
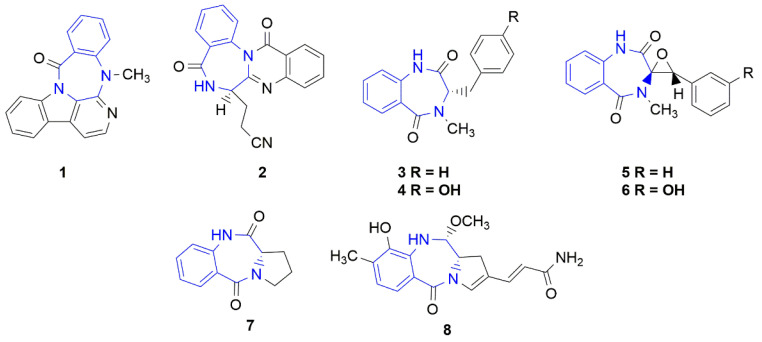
Benzodiazepine derivatives isolated from natural sources. The benzodiazepine core is marked in blue.

**Figure 2:**
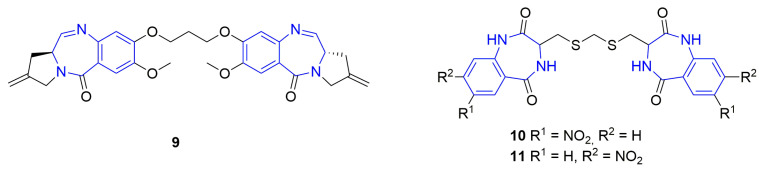
Dimeric benzodiazepines with anticancer activity. The benzodiazepine units are marked in blue.

Synthetic benzodiazepines and their analogues have found a wide range of applications as psychoactive [1] and anticancer [2,3] agents, and their biological activities have been further applied to other medical applications [4,5]. On the other hand, the naturally occurring seven-membered, heterocyclic benzodiazepine derivatives were rarely isolated from natural sources. The naturally occurring benzodiazepine derivatives could be divided into the following two main groups: benzodiazepine alkaloids or benzodiazepine dipeptides. The first group includes the pentacyclic carbazole alkaloid evodiagenine (**1**), which is isolated from the fruits of *Evodia rutaecarpa* (Juss.) Benth. [6] and exhibits cytotoxic activity against Sarcoma 180 and Heps (liver cancer) cell lines [7]. Another example is the tetracyclic quinazoline alkaloid auranthine (**2**), which is isolated from the mangrove endophytic fungus *Penicillium* 299# [8] (Figure 1). This compound showed no cytotoxic activities in vitro against various human cancer (MDA-MB-435, HepG2, HCT-116, and Calu-3) and normal IKHE cell lines [8,9]. The second group contains a series of bicyclic benzodiazepines **3**–**6**, which are isolated from various *Penicillium* strains and are composed of anthranilic acid and phenylalanine (tyrosine, 3-hydroxyphenylalanine) amino acid residues [10,11,12,13]. Cyclopeptine (3-benzyl-4-methyl-1,3-dihydro-1,4-benzodiazepine-2,5-dione) (**3**) and 14-hydroxy-cyclopeptine (**4**) have a simple, bicyclic structure, while cyclopenin (**5**) and cyclopenol (**6**) contain an additional epoxide ring within their structures. Another example is a large group of pyrrolo[1,4]benzodiazepine derivatives [14], such as cycloanthranilylproline (**7**), which is isolated from the plant *Isatis indigotica* [15] and the *Streptomyces cacaoi* strain [16] and is composed of anthranilic acid and proline. Cycloanthranilylproline (**7**) exhibited marked antileukemic activity against murine leukemia P388 cells in vitro with an IC_50_ value of 2.9 mg/mL [17]. The structure of the DNA minor groove alkylating agent anthramycin (**8**), which is isolated from the thermophilic actinomycete *Streptomyces refuineus*, is based on the molecular framework of cycloanthranilylproline (**7**) [14]. Anthramycin (**8**) exhibited significant anticancer properties and inhibited the growth of solid tumors in mice [18]. To reduce side effects, the original structure of anthramycin was subjected to further structural modifications [19], which led to the development of the medicinally relevant benzodiazepine dimer SJG-136 (NSC 694501, **9**, Figure 2), a rationally designed DNA minor groove interstrand cross-linking agent with a potent and broad spectrum of antitumor activity [20,21]. Finally, pyrrolo[1,4]benzodiazepine dimers were attached to tumor-targeting antibodies to create antibody–drug conjugates (ADCs) [22].

Our continuous research on the synthesis of medicinally relevant heterocyclic compounds based on the structure of dilactam [23,24,25,26,27,28,29,30], which could be treated as small, rigid, cyclic dipeptides with six-, seven-, or eight-membered dilactam rings, led to the discovery of tricyclic anthramycin analogs that exhibited selective antileukemic effects [23,24,28]. Our further research resulted in the development of novel tricyclic histone deacetylase (HDAC) inhibitors, which showed higher cytotoxicity and better selectivity towards leukemic and lymphoma cell lines when compared with the reference drug and HDAC inhibitor vorinostat [30]. Finally, the anticancer activity of proline-based diketopiperazines was the subject of our recent review [31]. Recently, we developed the synthesis of benzodiazepine dimers 10 and 11 (Figure 2), which possess nitro groups in their outer phenyl rings, where the benzodiazepine units are connected to each other with a five-membered linker containing three methylene groups and two sulfide bonds. The synthesized compounds 10 and 11 were tested on a panel of seven cancer and normal cell lines as potential cytotoxic and anticancer agents.

One of the mechanisms responsible for drug resistance in cancer is associated with the p53 protein. This protein product of the *TP53* tumor suppressor gene is a tetrameric nuclear transcription factor that suppresses cancer formation, activates DNA damage responses, arrests cell growth, and initiates apoptosis. Usually, cells lacking p53 are more resistant to chemotherapy, as in the case of cisplatin [32].

## 2. Materials and Methods

### 2.1. Chemistry

The commercially available chemicals were of reagent grade and used as received. L-djenkolic acid was purchased from Biosynth AG (Staad, Switzerland) and Santa Cruz Biotechnology, Inc. (Dallas, TX, USA). Next, 4-nitroisatoic anhydride and 5-nitroisatoic anhydride were ordered from Fluorochem Limited (Hadfield, UK). Potassium carbonate, dioxane, toluene, acetic acid, ethyl acetate, and acetone were purchased from Chempur (Piekary Śląskie, Poland). The reaction progress was monitored using LR-ESI-MS spectra, thin layer chromatography (TLC), and silica gel plates (Kieselgel 60F_254_, E. Merck, Darmstadt, Germany). The column chromatography that was used for the purification and isolation of the compounds was performed on silica gel 60 M (0.040–0.063 mm, E. Merck, Darmstadt, Germany). All ^1^H and ^13^C NMR spectra were recorded on a Bruker Avance III HD spectrometer operating at 500.20 (^1^H) and 125.79 (^13^C) MHz and equipped with a 5 mm probe head with Z-gradient coils. The experiments were performed using pulse programs from the standard Bruker library for samples dissolved in DMSO-*d_6_*. In each case, the spectra were calibrated at the residual solvent resonances. Multiplets were assigned as s (singlet), d (doublet), and m (multiplet). The Laboratory of Mass Spectrometry performed high-resolution mass spectra at the Institute of Biochemistry and Biophysics PAS on a LTQ Orbitrap Velos instrument from Thermo Scientific (Waltham, MA, USA). The spectra related to the synthesized compounds are included in the Supplementary Files.

#### General Procedure for the Synthesis of Dimeric Benzodiazepines **10** and **11**

The L-djenkolic acid **12** (1.00 g, 3.93 mmol, 1.0 equiv.) and appropriate 5-nitro **13** or 4-nitroisatoic anhydride **14** (1.72 g, 8.27 mmol, 2.1 equiv.) were dispersed in 50 mL of water:dioxane mixture (1:1 *v*/*v*), and potassium carbonate (1.63 g, 11.79 mmol, 3.0 equiv.) was added slowly in five portions. The reaction mixture was heated to 60 °C, and the temperature was maintained for 18 h. The volatiles were evaporated under reduced pressure, and the obtained solid was co-evaporated with toluene (3 × 50 mL). The residue was dissolved in 50 ml of acetic acid and heated under reflux for 18 h. The next day, the acetic acid was evaporated under reduced pressure, followed by co-evaporation with toluene (3 × 50 mL). The obtained solid was partitioned between water and ethyl acetate (100 mL:100 mL *v*/*v*), and the organic phase was washed with water (2 × 50 mL), a saturated solution of potassium carbonate in water (2 × 50 mL), and brine (1 × 50 mL). The organic phase was dried over anhydrous magnesium sulfate and filtrated and evaporated with silica gel. The products were purified and isolated using column chromatography on silica gel, pure ethyl acetate, ethyl acetate:acetone 7:3, and ethyl acetate:acetone 1:1 mixtures (*v*/*v*). The yield was 15–20%.

3,3′-((methylenebis(sulfanediyl))bis(methylene))bis(7-nitro-3,4-dihydro-1*H*-benzo[*e*][1,4]diazepine-2,5-dione) (**10**). The ratio of stereoisomers in the NMR spectrum was 54:46; ^1^H NMR (500 MHz, DMSO-*d*_6_) δ 11.06 (s, 2H, 2xNH), 8.86–8.76 (m, 2H, 2xNH), 8.55–8.47 (m, 2H, H_Ar_), 8.40–8.29 (m, 2H, H_Ar_), 7.30 (d, 2H, *J* = (9.0 Hz, H_Ar_), 4.09–3.98 (m, 2H, 2xCH), 3.75 (s, 2H, -S-CH_2_-S-), 3.09–2.99 (m, 2H, CH_2CH’’_), and 2.83–2.72 (m, 2H, CH_2CH’’_). ^13^C NMR (100 MHz, DMSO-*d*_6_) δ 170.65, 170.64, 165.1, 143.0, 142.1, 127.1, 127.0, 126.6, 126.2, 122.2, 51.93, 51.86, 35.1; 35.0, 28.4, and 28.3. HRMS (ESI): *m/z* [M + H]^+^ calculated for C_21_H_19_N_6_O_8_S_2_: 547.07003, found: 547.07069.

3,3′-((methylenebis(sulfanediyl))bis(methylene))bis(8-nitro-3,4-dihydro-1*H*-benzo[*e*][1,4]diazepine-2,5-dione) (**11**). The ratio of stereoisomers in the NMR spectrum was 58:42; ^1^H NMR (500 MHz, DMSO-*d*_6_) δ 10.81 (s, 2H, 2xNH), 8.89–8.76 (s, 2H, 2xNH), 8.05–7.90 (m, 6H, H_Ar_), 4.05–3.95 (m, 2H, 2xCH), 3.73 (s, 2H, -S-CH_2_-S-), 3.08–2.98 (m, 2H, CH_2CH″_), and 2.81–2.71 (m, 2H, CH_2CH′_). ^13^C NMR (100 MHz, DMSO-*d*_6_) δ 170.85, 170.83, 166.2, 149.34, 149.33, 137.6, 132.62, 132.59, 131.35, 131.34, 118.1, 116.0, 115.9, 51.8, 35.2, 35.1, 28.5, and 28.4. HRMS (ESI): *m/z* [M + H]^+^ calculated for C_21_H_19_N_6_O_8_S_2_: 547.07003, found: 547.07034.

### 2.2. Biology

#### 2.2.1. Cell Lines and Culture Conditions

The following cell lines were used in this study: epidermoid carcinoma A-431 (ATCC^®^ CRL-1555), lung carcinomas A549 (ATCC^®^ CCL-185) and NCI-H1299 (ATCC^®^ CRL-5803), colorectal carcinomas HCT116 (ATCC^®^ CCL-247) and HCT116p53^−/−^, normal skin fibroblasts BJ (ATCC^®^ CRL-2522), and normal lung fibroblasts MRC-5 (ATCC^®^ CCL-171). The HCT116 p53^−/−^ cell line was a kind gift from Professor Bert Vogelstein (John Hopkin’s University, Baltimore, MD, USA). The remaining cell lines were purchased from ATCC (Manassas, VA, USA). The cells were cultured in minimal, Dulbecco’s modified Eagle’s, or Ham’s F-12K medium supplemented with 10% fetal bovine serum (Gibco by Thermo Fisher Scientific, Waltham, MA, USA) and grown under standard culture conditions (37°C, 5% CO_2_) in a humidified incubator with an oxygen concentration of 21%. All of the experiments were performed with mycoplasma-free cells.

#### 2.2.2. Cell Viability Assay

The viability of the cells was assayed by measuring the conversion of MTT (3-(4,5-dimethylthiazol-2-yl)-2,5-diphenyltetrazolium bromide) to formazan (the rate of this reaction is proportional to the number of surviving cells). The cells were seeded in 96-well culture plates at a density of 3500–5000 cells per well 24 h before the treatment. Six wells were considered for each experimental point (technical repeats). Treatments with increasing concentrations of **10**, **11**, or cisplatin (0.6–80 μM) were performed for 72 h. The MTT stock solution (Sigma-Aldrich by Merck, Darmstadt, Germany) was added at a final concentration of 0.5 mg/mL. After 4 h of incubation at 37 °C, the water-insoluble formazan was dissolved in a lysis buffer containing 20% SDS, 50% DMF, 2.5% hydrochloric acid, and 2.5% acetic acid. The optical densities were measured at 570 nm using a scanning multi-well spectrophotometer (PARADIGM Detection Platform; Beckman Coulter, Brea, CA, USA). The ratio of the absorbance value of each tested group to the absorbance value of the untreated control was calculated, and each tested group’s cell viability was expressed as a percentage of the control. The cell viability assays were performed at least in triplicate using independent cell cultures.

#### 2.2.3. Cell Proliferation and Death Assay

The cell proliferation and death were estimated in the IncuCyte S3 live-cell imaging system using the SYTOX™ Green Dead Cell Stain (Thermo, cat. no: S34860) and analyzed using the IncuCyte 2019B Rev2 software (Essen BioScience, Ann Arbor, MI, USA), according to the manufacturer’s instructions (https://www.sartorius.com/en/products/live-cell-imaging-analysis/live-cell-analysis-resources/live-cell-imaging-and-analsis-handbook (accessed on 21 October 2022)). The HCT116 and HCT116 p53^−/−^ cells were seeded in 96-well culture plates at a density of 5000 cells per well 24 h before the treatment. Four wells were considered for each experimental point. Treatments with **10**, **11**, cisplatin (40 μM), or dimethyl sulfoxide (which was used as a negative control at a concentration equal to the one used to dissolve **10** and **11**) were carried out for up to 72 h, and images were recorded every 2 h during this time. The experiment was performed twice.

#### 2.2.4. Statistical Analysis

The data are presented as means ± SDs. The differences among the variables were analyzed using a Student’s *t*-test. The differences among the groups were evaluated using a one-way analysis of variance (ANOVA), followed by a post-hoc Tukey’s test. The differences were considered significant if the *p*-value was ≤ 0.05. All of the statistical analyses were performed using the Statistica software package (StatSoft, Tulsa, OK, USA).

## 3. Results and Discussion

### 3.1. Chemistry

**Scheme 1:**
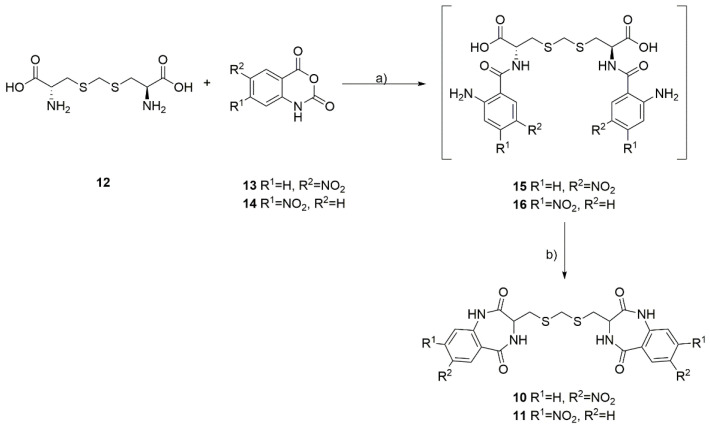
Synthesis of dimeric benzodiazepines **10** and **11** from L-djenkolic acid (**12**) and nitroisatoic anhydrides **13** and **14**. (**a**) K_2_CO_3_, dioxane:H_2_O 1:1, 60 °C, 18 h; (**b**) AcOH, rfx, 18 h.

The developed synthesis of the dimeric dibenzodiazepines **10** and **11** utilizes a commercially available, naturally occurring, and non-proteogenic amino acid, known as djenkolic acid (**12**), which is isolated from legume seeds. In the first step, the djenkolic acid (**12**) was reacted with 5-nitroisatoic anhydride (**13**) or 4-nitroisatoic anhydride (**14**) in the presence of potassium carbonate, which led to the formation of the linear tripeptides **15** and **16** (Scheme 1). Next, we attempted a double cyclization of the obtained linear tripeptides using the classical methods of amide bond formation used in peptide synthesis. Surprisingly, using either carbodiimides (such as EDC) or uronium salts (such as HATU) to activate the carboxyl group failed to identify the desired products in the reaction mixture. We observed that only heating the linear **15** and **16** tripeptides in boiling acetic acid for 18 h proved to be an effective method for double cyclization, leading to the formation of two intramolecular amide bonds. The desired products were isolated with low yields and purified by column chromatography, and NMR spectra confirmed their purity and structure.

From the obtained results, we assumed that the linear tripeptides **15** and **16** are very difficult to undergo double cyclization to the corresponding benzodiazepine dimers and that relatively harsh dehydration conditions are needed to obtain **10** and **11**. Unfortunately, the analysis of the NMR spectra revealed that the synthesized compounds exist in two forms in a ~1:1 ratio, and the ratio of these two forms does not depend on the measurement temperature of the NMR spectra or the degree of dilution of the sample. In this case, we excluded the observation of compound conformers and the formation of inter- and intramolecular interactions stabilizing specific forms of the compound in the solution. We assumed that in the final stage of the reaction, racemization of the amino acid occurred, which led to the formation of a mixture of stereoisomers of the desired products. So far, all attempts to obtain optically pure products have failed, and this is the subject of our ongoing research.

### 3.2. Biology

Using cisplatin as a reference anticancer drug, the synthesized compounds **10** and **11** were evaluated in vitro for their cytotoxic activities, including their effects on cell viability, proliferation, and cell death.

#### 3.2.1. Cancer Cells Are Hypersensitive to **10** and **11**

**Figure 3:**
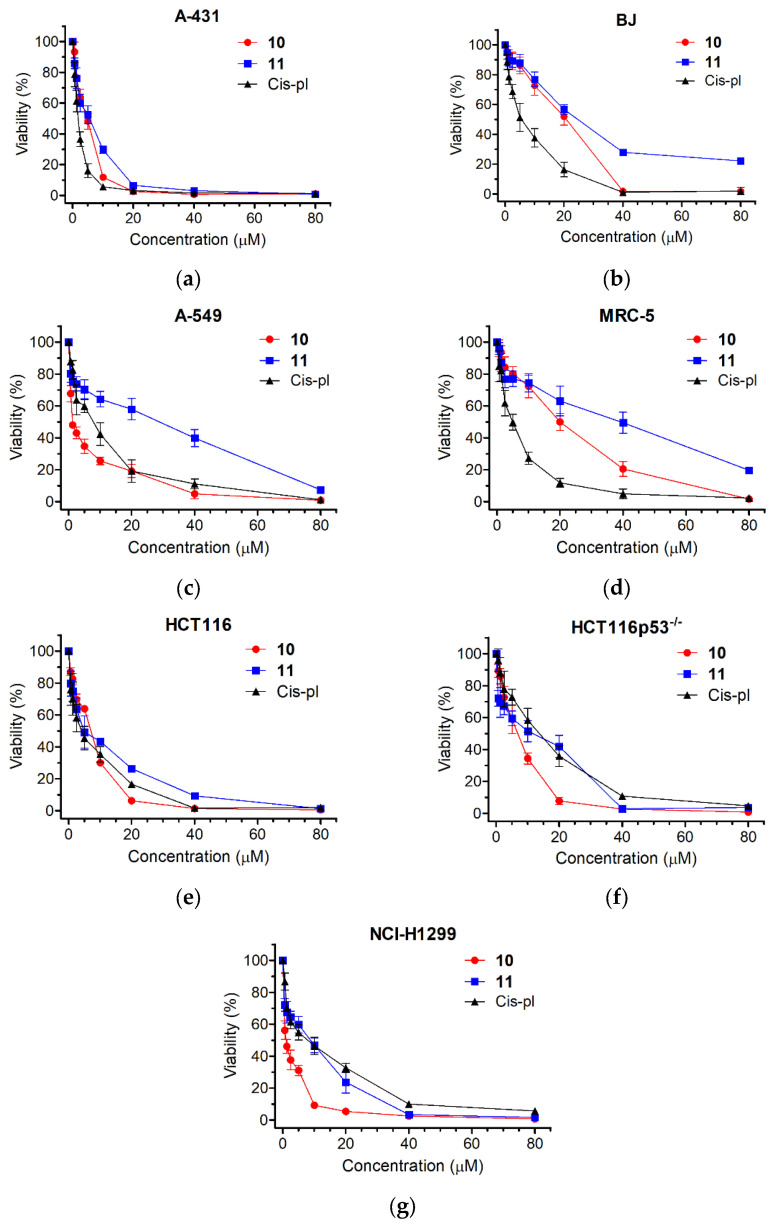
Viability of selected human cell lines in response to compounds **10**, **11**, or cisplatin (cis-pl), evaluated with an MTT assay. The cell lines studied were as follows: (**a**) epidermoid carcinoma A-431, (**b**) normal skin fibroblasts BJ, (**c**) lung carcinoma A549, (**d**) normal lung fibroblasts MRC-5, (**e**,**f**) colorectal carcinomas HCT116 and HCT116p53^−/−^, and (**g**) lung carcinoma NCI-H1299. The cells were incubated with increasing concentrations (range = 0.6–80 µM) of compounds **10**, **11**, or cis-pl for 72 h. Cisplatin, a well-established DNA damage agent commonly used as a drug in chemotherapy, served as a positive control. The percentage of cell viability inhibition was calculated compared to the untreated cells. Each point on the curves represents the mean ± SD of at least three independent experiments performed in six replicates per drug concentration.

Compounds **10** and **11** were tested using an MTT metabolic assay to determine their potential ability to inhibit the viability of the following five cancer-derived cell lines: epidermoid carcinoma A-431, lung carcinomas A549 and NCI-H1299, colorectal carcinomas HCT116 and HCT116p53^−/−^ (an isogenic cell line to HCT116 with a *TP53* gene knockout), and the following two non-cancer derived cell lines: normal skin fibroblasts BJ (a reference line to A-431) and lung fibroblasts MRC-5 (a reference line to A549 and NCI-H1299). A549 cells are used as models for studying lung cancer and developing drug therapies against it. HCT116 is also a cell line used in therapeutic research and drug screenings. The viability results and obtained half-maximal viability inhibitory concentrations (IC_50_) are shown in Figure 3 and Table 1. The cytotoxicity of each derivative differed depending on the dose and type of the treated cell line. The highest toxicity was observed for **10** in the lung cancer cell lines. The IC_50_ values varied from 1.07 ± 0.25 μM (NCI-H1299) to 1.6 ± 0.21 μM (A549) and were much lower than in the case of cisplatin (5.7 ± 0.88 μM, *p* < 0.001 for NCI-H1299 and 5.95 ± 1.28 μM, *p* < 0.01 for A549). Thus, compound **10** was 5.33 times more cytotoxic for the NCI-H1299 cell line than cisplatin and 3.72 times more cytotoxic for the A549 cell line than cisplatin. The least cytotoxic effect was observed for the normal cell lines, BJ and MRC-5, where the IC_50_ values were, respectively, 16.72 ± 2.77 μM and 16.85 ± 2.96 μM for **10** and 23.1 ± 2.34 and 28.7 ± 7.6 μM for **11**. Interestingly, the IC_50_ values of the BJ and MRC-5 cells exposed to cisplatin were significantly higher compared to the treatments with **10** or **11** (5.06 ± 0.94, *p* < 0.01 or 0.001 and 4.17 ± 0.88, *p* < 0.01, respectively).

The cytotoxic effect obtained for the two lung cancer cell lines was then compared with the cytotoxic effect obtained for the reference normal cell line MRC-5. Cisplatin exhibited very low selectivity indices, which are as follows: 0.74 ± 0.3 for A549 and 0.75 ± 0.22 for NCI-H1299, respectively. Contrary to cisplatin, compound **10** showed very high selectivity indices, which are as follows: 10.54 ± 1.54, *p* < 0.001 for A549 and 16.08 ± 3.43, *p* < 0.001 for NCI-H1299, respectively (Table 1). Also, when tested on the NCI-H1299 cancer and normal lung (MRC-5) cells and on the cancer (A-431) and normal (BJ) skin cell lines, **11** exhibited a higher selectivity index than cisplatin (6.42 ± 2.95 vs. 0.75 ± 0.22, *p* < 0.05 and 5.89 ± 0.58 vs. 3.26 ± 0.41, *p* < 0.01, respectively; Table 1). Overall, the results demonstrate that both compounds could be considered candidates for selective anticancer treatments.

The ability to avoid growth inhibition is a characteristic ability to maintain proliferative signals in cancer cells. There are several genes encoding tumor-suppressive proteins, among them *TP53*, which act in different ways to prevent cell growth and proliferation [33]. Although the frequency varies according to the type of cancer, *TP53* mutations are present in approximately 50% of all malignancies [34]. Usually, cells lacking p53 are more resistant to chemotherapy, as in the case of cisplatin and the two isogenic colon cancer lines HCT116 and HCT116p53^−/−^ (Figure 3e,f and Table 1). The HCT116p53^−/−^ cells were 3.07 times more resistant to cisplatin than HCT116 (IC_50_ = 10.77 ± 2.86 μM for HCT116p53^−/−^ and 3.51 ± 1.16 μM for HCT116, *p* < 0.05). Contrary to cisplatin, the cytotoxic effects of **10** and **11** were not dependent on p53 and were comparable for both the HCT116 and HCT116p53^−/−^ cell lines (IC_50_ = 5.31 ± 0.23 μM and 5.56 ± 0.74 μM, *p* = 0.61 for **10** and 4.9 ± 0.53 μM and 6.07 ± 0.9, *p* = 0.12 for **11**, respectively; Figure 3e,f and Table 1). We also observed that the NCI-H1299 cell line, which has a homozygous partial deletion of the *TP53* gene and, as a result, does not express the tumor suppressor p53 protein (that in part accounts for their proliferative propensity), was more sensitive to **10** than to cisplatin (1.07 ± 0.25 vs. 5.7 ± 0.88, *p* < 0.001; Figure 3g and Table 1), confirming the higher specificity of **10** towards cells that lack p53.

#### 3.2.2. Treatment with **10** and **11** Inhibits Proliferation and Induces Massive Cell Death Independent of p53 Background

**Figure 4:**
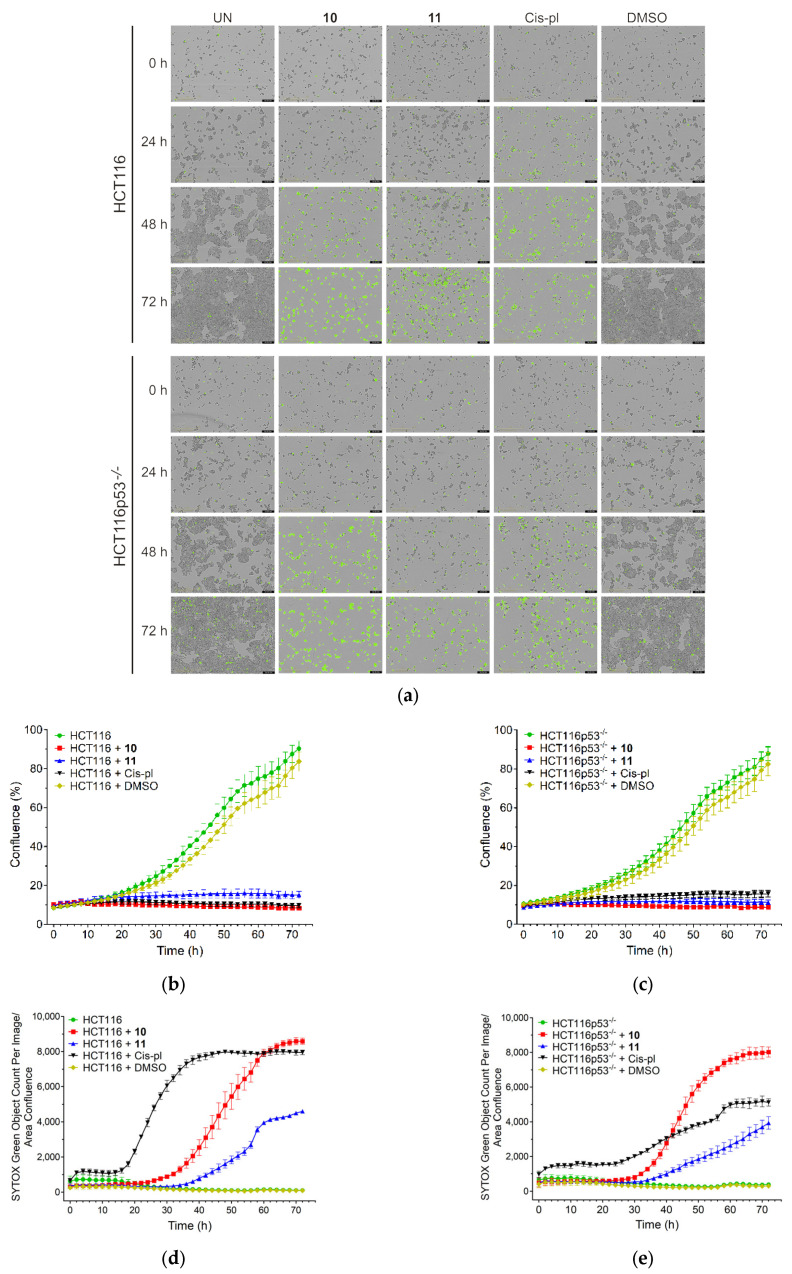

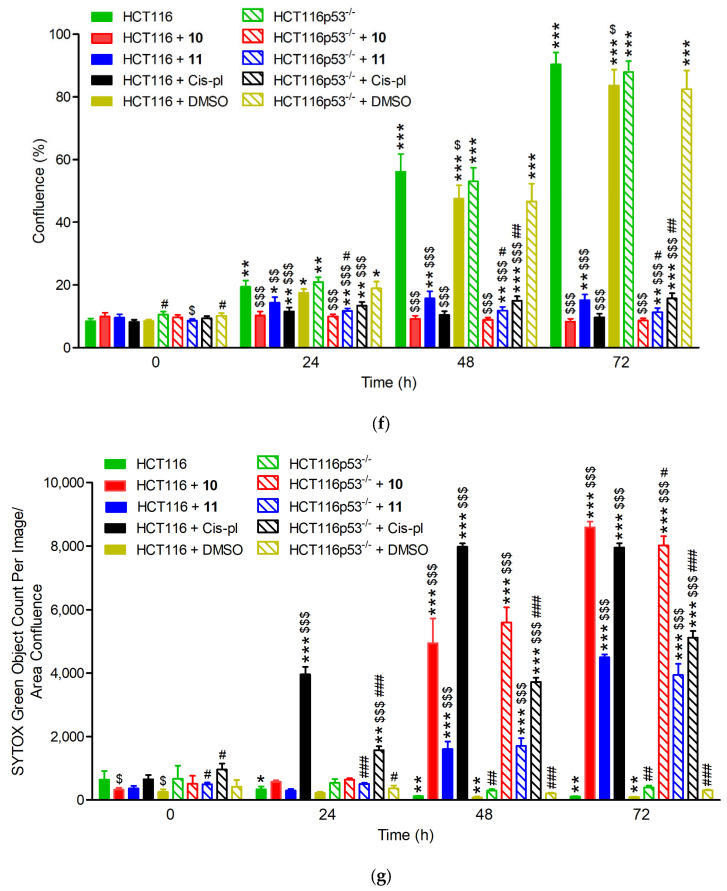
Confluence and cell death measured by IncuCyte live-cell imaging in wild-type (HCT116) and p53 null mutant (HCT116p53^−/−^) cell lines treated with 40 µM of **10**, **11**, and cisplatin (cis-pl), or dimethyl sulfoxide (DMSO), which was used as a negative control. (**a**) Representative images from the live-cell imaging at 0, 24, 48, and 72 h with or without a drug (UN). The yellow scale bars on the images in the lower left corner represent 400 μm. The confluence was drastically reduced and the cell death (measured with SYTOX Green Dead Cell Stain and calculated as the percentage of the total cell population) was significantly increased in the HCT116 (**b**,**d**) and HCT116p53^−/−^ cells (**c**,**e**) after **10, 11**, or cis-pl treatment compared with the untreated or DMSO-treated cells. (**f**,**g**) Analysis of confluence and cell death after treatment for 24, 48, and 72 h with 40 µM of **10**, **11**, and cis-pl, or DMSO. Data are presented as means ± SDs of four replicative wells. The statistical analysis was performed using one-way ANOVA, followed by the post-hoc Tukey’s test (*, *p* < 0.05; **, *p* < 0.01; ***, *p* < 0.001 treated cells vs. corresponding untreated cells in time 0 h; $, *p* < 0.05; $$, *p* < 0.01; $$$, *p* < 0.001 treated cells vs. corresponding untreated cells in the same time) or two-tailed Student’s *t*-test (#, *p* < 0.05; ##, *p* < 0.01; ###, *p* < 0.001 HCT116p53^−/−^ cells vs. corresponding HCT116 cells).

The sensitivity of the HCT116 and HCT116p53^−/−^ cell lines to compounds **10** and **11** was also analyzed by IncuCyte live-cell imaging. This was performed to reveal the rate of cell proliferation and death in the p53-proficient and deficient background. The cells were cultured with 40 µM concentrations of **10**, **11**, and cisplatin and filmed once every 2 h for 72 h using the IncuCyte system. When cultured without drugs or with dimethyl sulfoxide (DMSO), which was used as a negative control, the HCT116 and HCT116p53^−/−^ cells grew to near full confluence, reaching an average of 90% or 84% and 88% or 83% confluence, respectively, on the third day. When cultured in the presence of 40 µM of drugs, a drastic reduction in the confluence occurred in both cell lines, reaching only up to 16% confluence (*p* < 0.001 for all drugs; Figure 4a–c, f and Supplementary Figure S9).

The effects of **10**, **11**, or cisplatin on cell viability were confirmed with the SYTOX Green Dead Cell Stain, which visualizes dead cells irrespective of the death mode (Figure 4a). There was no increase in cell death in the untreated or DMSO-treated cells. The rate of cell death after treatment with **10** or **11** was similar in HCT116 and HCT116p53^−/−^ (only slightly lower in p53-deficient cells exposed to **10** for 72, *p* < 0.05, Figure 4g) and significantly increased starting at 24 or 36 h for **10** and **11**, respectively. Thus, treatment for up to 1–1.5 days has a cytostatic effect, inhibiting proliferation only; however, prolonged incubation with each compound led to massive cell death, which reached a plateau on the third day for **10** (Figure 4a,d,e,g).

The death rate of the cisplatin-exposed cells was different in the p53-deficient cells compared to the proficient ones (Figure 4a,d,e,g). In the HCT116 cell line, a huge increase in the number of SYTOX Green positive cells appeared 18 h after the treatment and reached its highest value at 48 h. The course of the cell death curve in the HCT116p53^−/−^ line was much flatter, which indicates much weaker dynamics of cell death in this cell line, reaching a plateau after about 72 h at a much lower level than in the HCT116 cells (5116.3 ± 211.41 vs. 7949.12 ± 134.73, *p* < 0.001; Figure 4d,e,g). These results are consistent with the literature data, showing that cisplatin-induced cell death via apoptosis is p53-dependent [35,36]. The ability of p53 to induce the apoptosis of cells exposed to environmental or oncogenic stress constitutes a major pathway, whereby p53 exerts its tumor suppressor functions [32]. As in cancer cells, the *TP53* tumor suppressor gene is inactivated more often than in any others, our results may provide the basis for constructing chemotherapeutic agents that are more effective and cause fewer adverse effects.

## 4. Conclusions

During our studies, we identified compounds based on a unique, dimeric benzodiazepine scaffold that exhibited exceptional anticancer effects against various cell lines and were superior to the reference cancer drug cisplatin in the following three ways: (i) compound **10** showed a much stronger cytotoxic effect on the lung cancer cell lines (A549 and NCI-H1299) than cisplatin; (ii) the calculated selectivity indices for compounds **10** and **11**, obtained by comparing the values for the tumor and normal lines, were, in most cases, higher than those for cisplatin; (iii) contrary to cisplatin, the observed cytotoxic effect was not dependent on the p53 protein, which is one of the factors responsible for the drug resistance of tumors. We would emphasize that the synthesized products are low-molecular-weight compounds with lead-like properties. The applied molecular framework could serve as a convenient privileged structure for designing and developing novel bioactive molecules suitable for drug design, development, and optimization. The results suggest that this class of compounds could be applied to the treatment of lung cancers.

## Figures and Tables

**Table 1:** Calculated IC_50_ values of compounds **10**, **11**, and cisplatin on selected human cell lines in vitro.

Compound No.	10	11	Cisplatin
	IC_50_ (µM) ^a^		
A-431 (epidermoid cancer) BJ (normal skin) SI index: IC_50_ BJ/IC_50_ A-431	3.47 ± 0.32 (**) ^b^	3.97 ± 0.87 (**) ^b^	1.56 ± 0.29
	16.72 ± 2.77 (**) ^b^	23.1 ± 2.34 (***) ^b^	5.06 ± 0.94
	4.88 ± 1.08 (**) ^c^ (ns) ^b^	5.89 ± 0.58 (**) ^c^ (**) ^b^	3.26 ± 0.41 (**) ^c^
A549 (lung cancer) MRC-5 (normal lung) SI index: IC_50_ MRC-5/IC_50_ A549	1.6 ± 0.21 (**) ^b^	16.29 ± 4.65 (*) ^b^	5.95 ± 1.28
	16.86 ± 2.96 (**) ^b^	28.7 ± 7.6 (**) ^b^	4.17 ± 0.88
	10.54 ± 1.54 (***) ^c^ (***) ^b^	1.86 ± 0.79 (ns) ^c^ (ns) ^b^	0.74 ± 0.3 (ns) ^c^
NCI-H1299 (lung cancer) MRC-5 (normal lung) SI index: IC_50_ MRC-5/IC_50_ NCI-H1299	1.07 ± 0.25 (***) ^b^	4.91 ± 1.45 (ns) ^b^	5.7 ± 0.88
	16.86 ± 2.96 (**) ^b^	28.7 ± 7.6 (**) ^b^	4.17 ± 0.88
	16.08 ± 3.43 (***) ^c^ (**) ^b^	6.42 ± 2.95 (**) ^c^ (*) ^b^	0.75 ± 0.22 (ns) ^c^
HCT116 (colon cancer) HCT116p53^−/−^ (colon cancer) SI index: IC_50_ HCT116p53^−/−^/IC_50_ HCT116	5.31 ± 0.23 (*) ^b^	4.9 ± 0.53 (ns) ^b^	3.51 ± 1.16
	5.56 ± 0.74 (*) ^b^	6.07 ± 0.9 (*) ^b^	10.77 ± 2.86
	1.05 ± 0.19 (ns) ^c^ (*) ^b^	1.26 ± 0.3 (ns) ^c^ (*) ^b^	3.18 ± 0.79 (*) ^c^

^a^ Data are expressed as means ± SDs, n = 3; IC_50_, the lethal dose for 50% of cells; SI, selectivity index. ^b^ Stars indicate a statistically significant difference in the IC_50_ values or SI index between **10** or **11** and cisplatin. ^c^ Stars indicate a statistically significant difference in the IC_50_ values between the cancer and normal cell lines or HCT116 and HCT116p53^−/−^. The statistical analysis was performed using Student’s *t*-test; *, *p* < 0.05 **, *p* < 0.01; ***, *p* < 0.001; ns, non-significant.

## Data Availability

Not applicable.

## Supplementary Materials

The following supporting information can be downloaded at: https://www.mdpi.com/article/10.3390/biom13020291/s1, Figure S1. HRMS spectrum of **10**; Figure S2. HRMS spectrum of **12**; Figure S3. ^1^H NMR spectrum of **10**; Figure S4. ^13^C NMR spectrum of **10**; Figure S5. Dept135 NMR spectrum of **10**; Figure S6. ^1^H NMR spectrum of **11**; Figure S7. ^13^C NMR spectrum of **11**; Figure S8. Dept135 NMR spectrum of **11**; Figure S9. Confluence measured by IncuCyte live-cell imaging in wild-type (HCT116) and p53 null mutant (HCT116p53-/-) cell lines treated with 40 µM of **10**, **11**, cisplatin (cis-pl), or dimethyl sulfoxide (DMSO), which was used as a negative control.
