# Supplementary material for: Dimeric Benzodiazepines as Peptide Mimetics to Overcome p53-Dependent Drug Resistance of Tumors
Source: Biomolecules. 2023 Feb 3;13(2):291. doi: 10.3390/biom13020291 (PMC9953746; doi:10.3390/biom13020291)
Supplement: Supplementary file 1 [file biomolecules-13-00291-s001.zip › biomolecules-2132462-supplementary.pdf]

Supplementary Materials

Dimeric Benzodiazepines as Peptide Mimetics To Overcome p53-dependent Drug Resistance of Tumors

Elżbieta Speina [1], Marcin Wilczek [2], Adam Mieczkowski [1,*]

[1] Institute of Biochemistry and Biophysics Polish Academy of Sciences 5a, Pawinskiego 5a, 02-106 Warsaw, Poland
[2] Department of Chemistry, University of Warsaw, Pasteura 1, 02-093, Warsaw, Poland
* Correspondence: amiecz@ibb.waw.pl

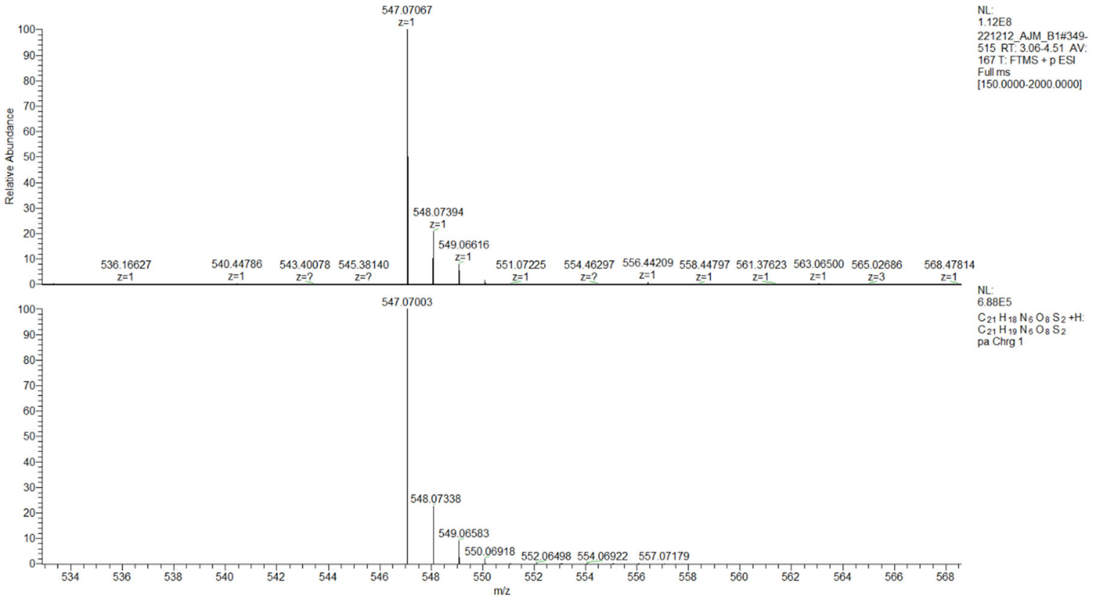

Figure S1. HRMS spectrum of **10**.

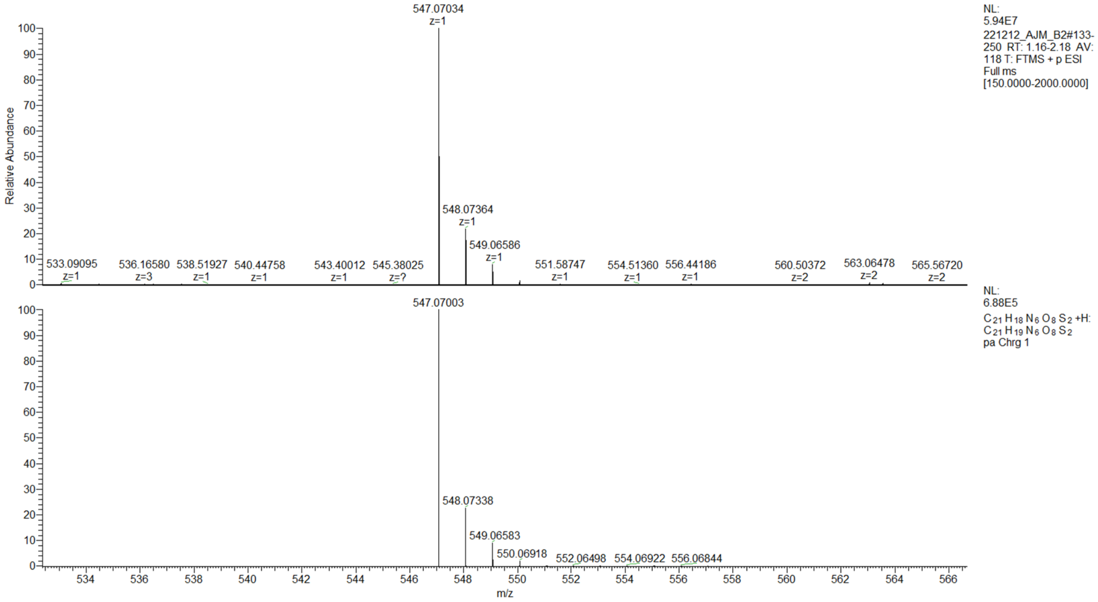

Figure S2. HRMS spectrum of **11**.

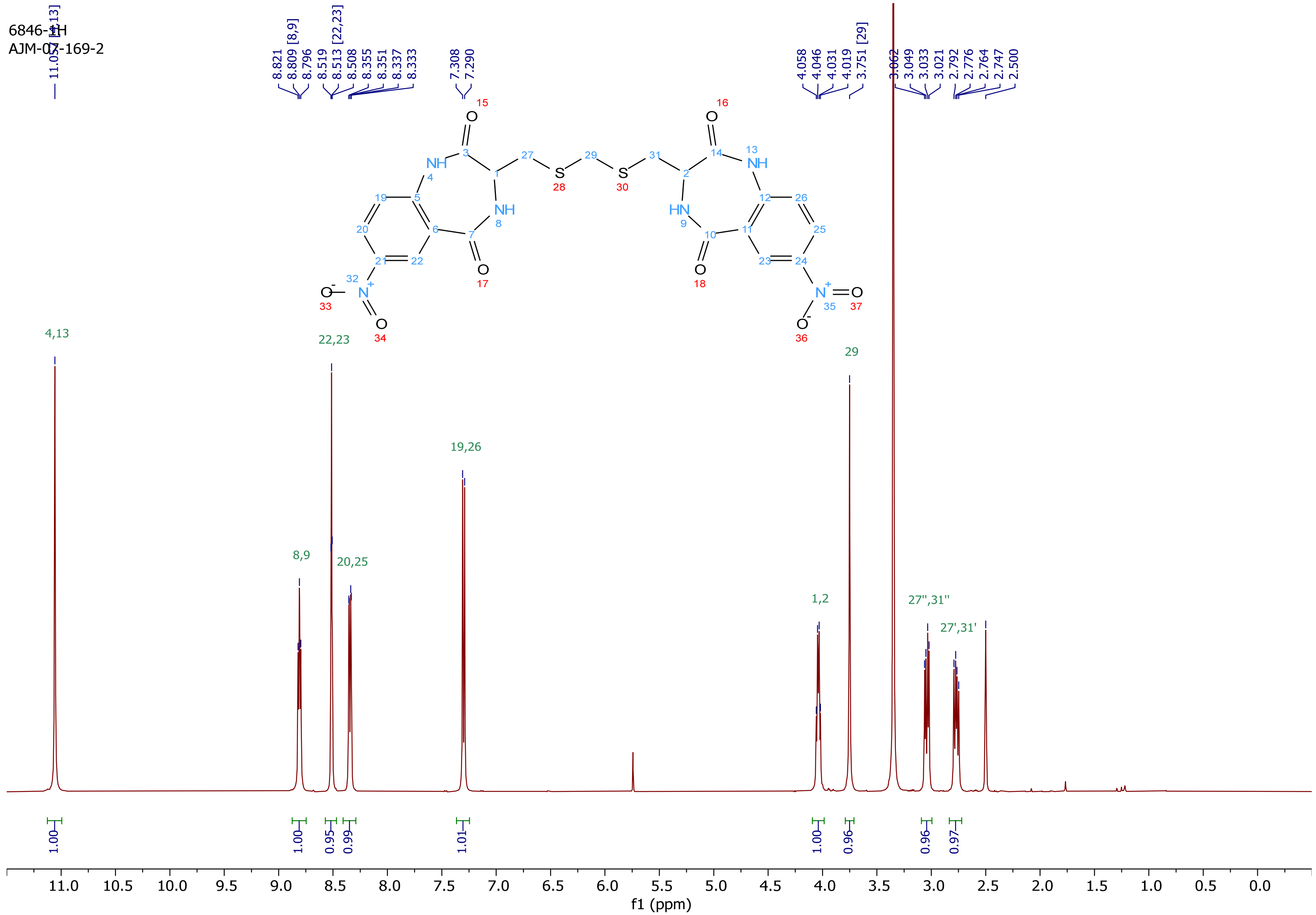

Figure S3. ^{1}H NMR spectrum of **10**.

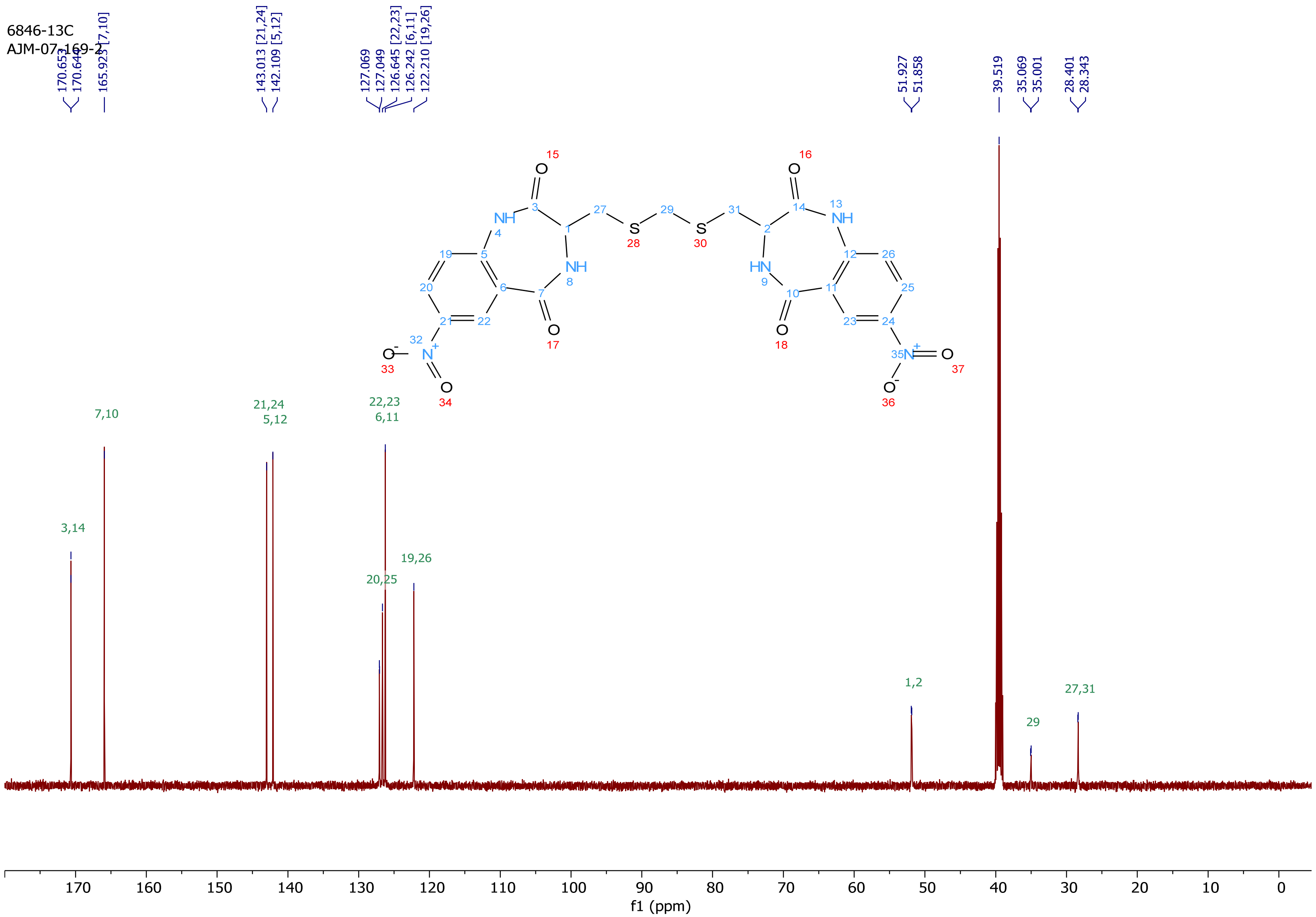

Figure S4. ^{13}C NMR spectrum of **10**.

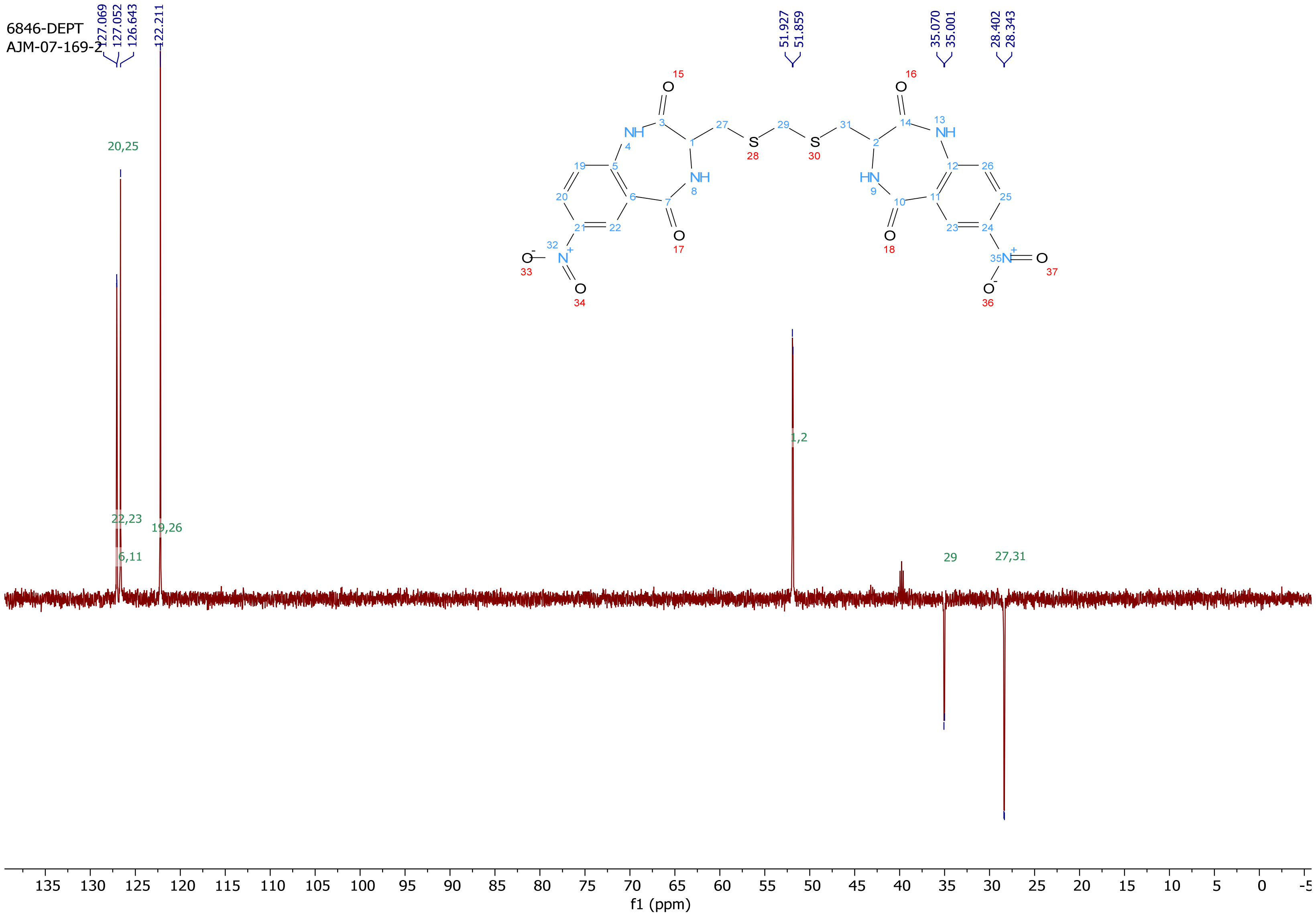

Figure S5. Dept135 NMR spectrum of **10**.

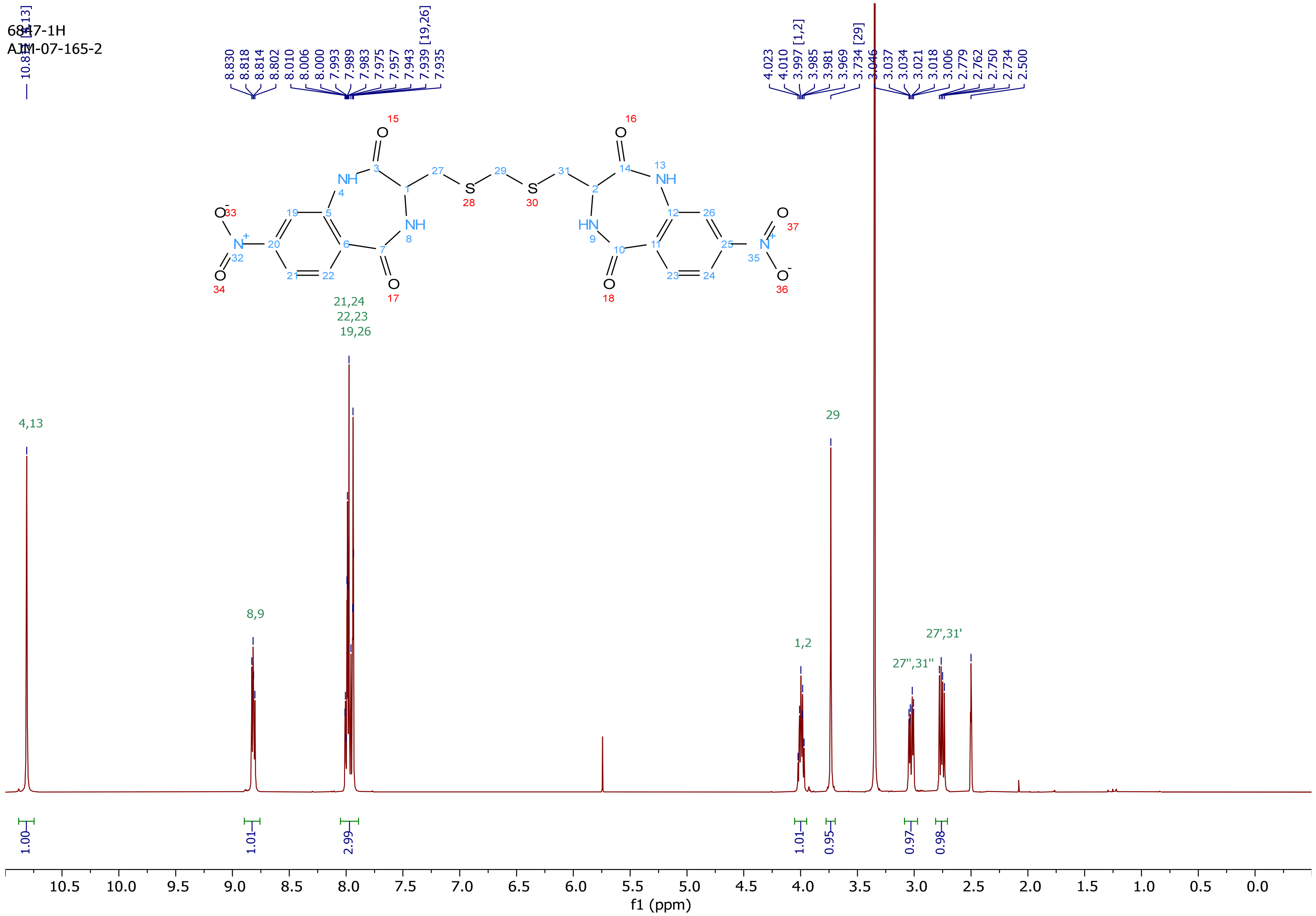

Figure S6. ^{1}H NMR spectrum of **11**.

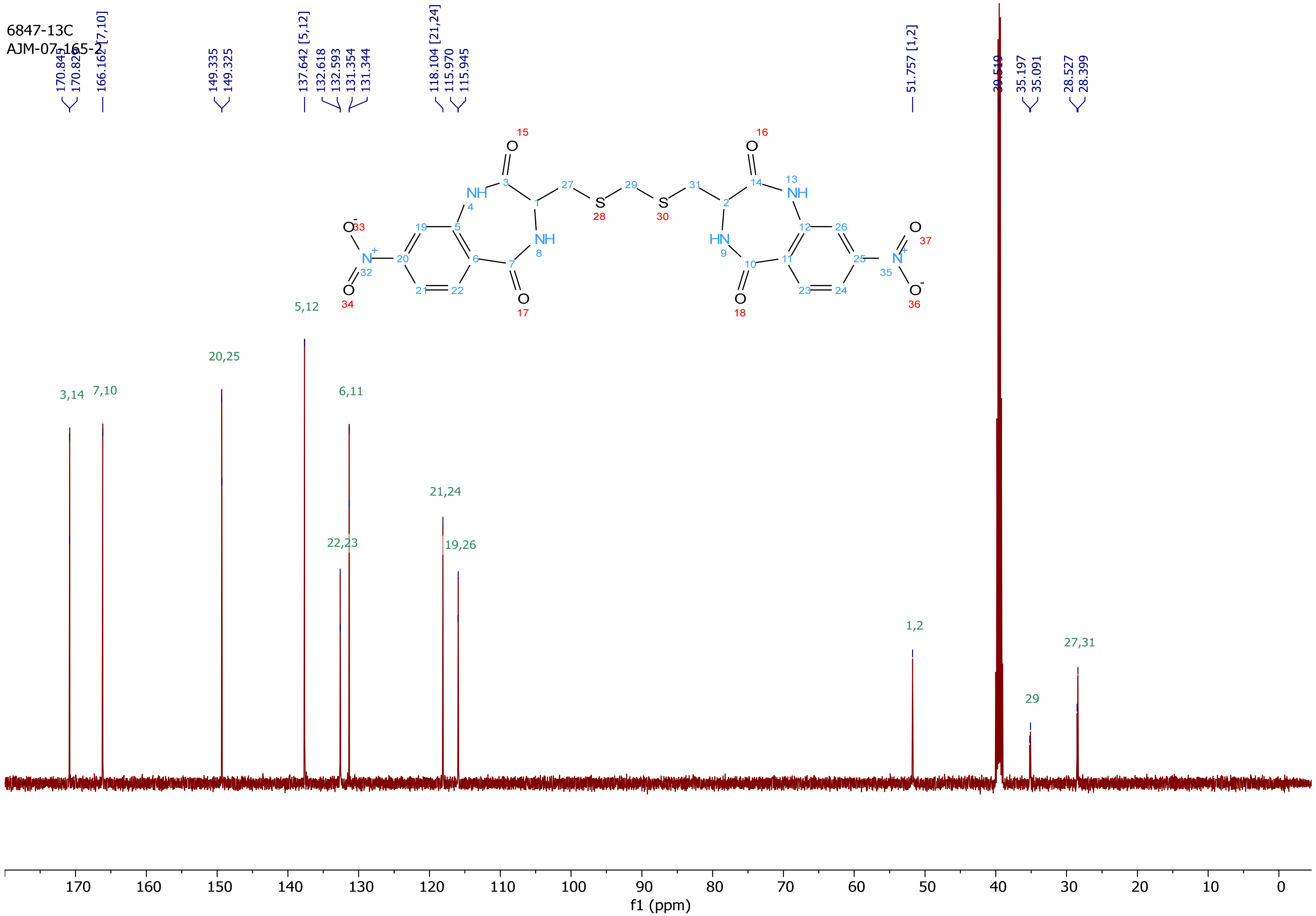

Figure S7. ^{13}C NMR spectrum of **11**.

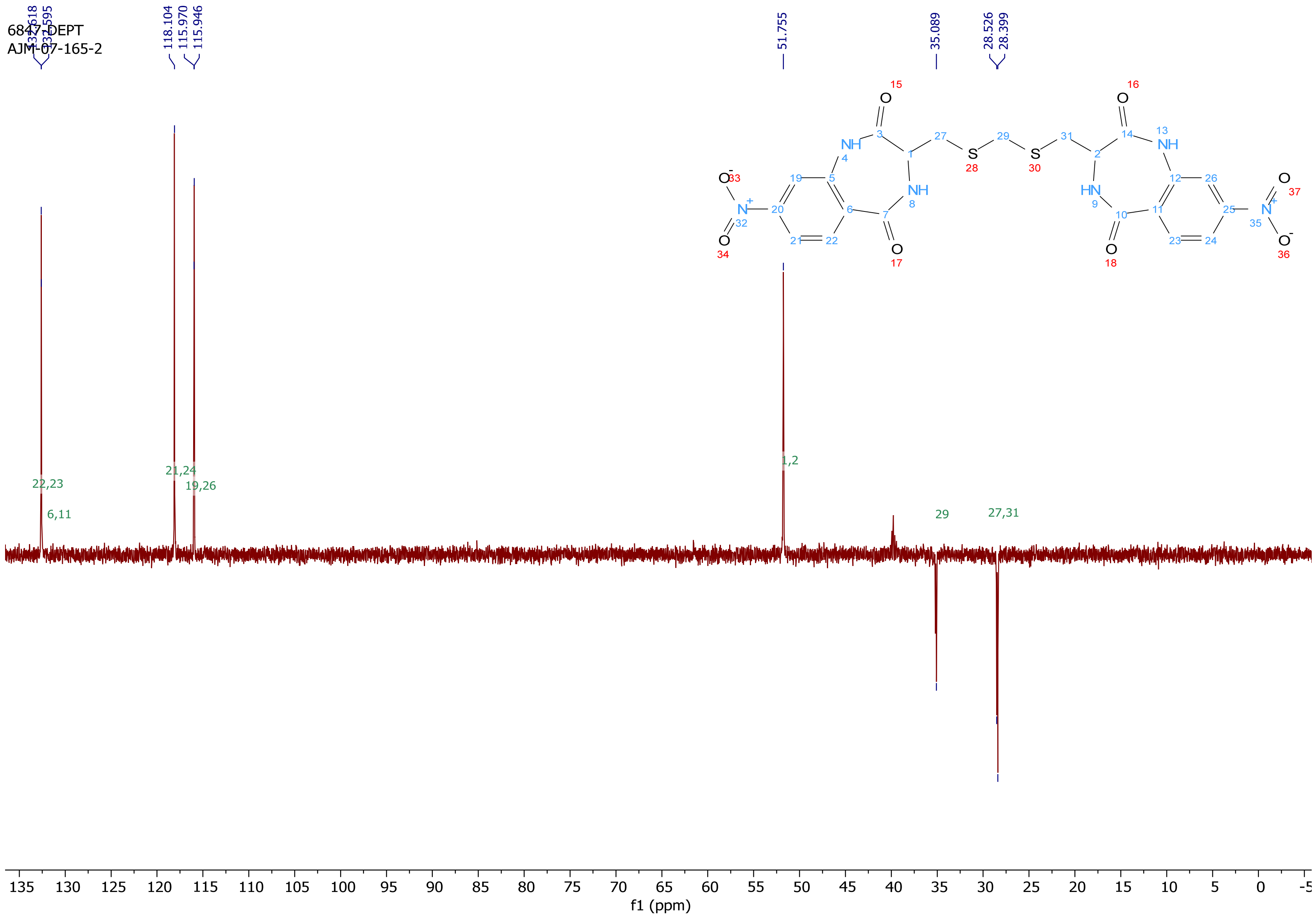

Figure S8. Dept135 NMR spectrum of **11**.

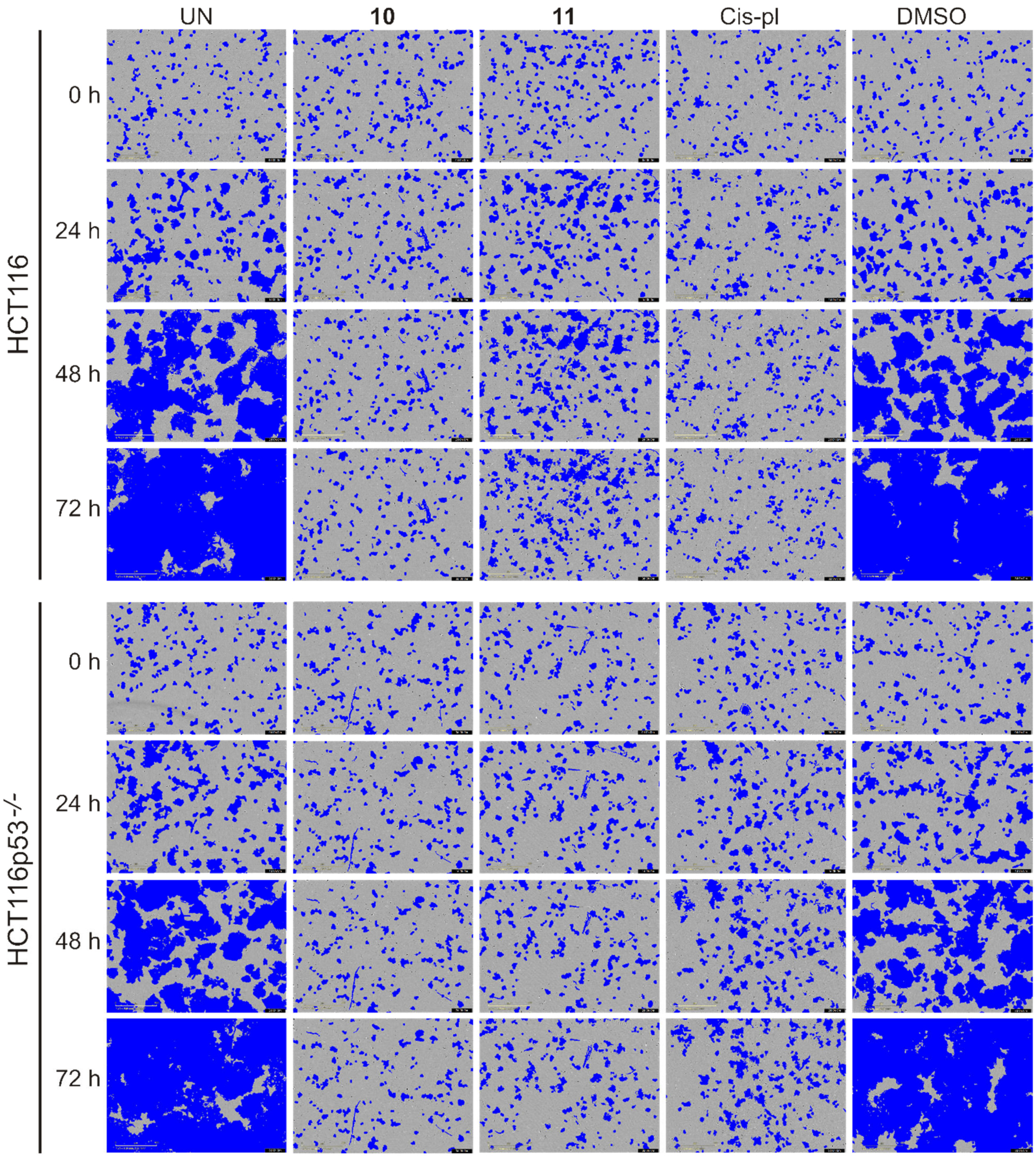

Figure S9. Confluence measured by IncuCyte live-cell imaging in wild-type (HCT116) and p53 null mutant (HCT116p53$^{-/-}$) cell lines treated with 40 μM **10**, **11**, cisplatin (cis-pl) or dimethyl sulfoxide (DMSO), used as a negative control. Representative images showing confluence (in blue) at 0, 24, 48 and 72 h with or without a drug are shown. UN, untreated cells. Yellow scale bars on images in the lower left corner represent 400 μm.
